# Opioid Prescription in Patients With Chronic Kidney Disease: A Systematic Review of Comparing Safety and Efficacy of Opioid Use in Chronic Kidney Disease Patients

**DOI:** 10.7759/cureus.45485

**Published:** 2023-09-18

**Authors:** Victor A Odoma, Aakanksha Pitliya, Esraa AlEdani, Japneet Bhangu, Khalid Javed, Prabhleen Kaur Manshahia, Shamsun Nahar, Srishti Kanda, Uzair Chatha, Lubna Mohammed

**Affiliations:** 1 Cardiology/Oncology, Indiana University Health, Bloomington, USA; 2 Internal Medicine, Sri Aurobindo Institute of Medical Science, Indore, IND; 3 Internal Medicine, California Institute of Behavioral Neurosciences & Psychology, Fairfield, USA; 4 Anesthesiology, Internal Medicine, St. George's University School of Medicine, Chicago, USA; 5 Medicine, All India Institute of Medical Sciences, Rishikesh, IND; 6 Internal Medicine, JC Medical Institute, Orlando, USA; 7 Medicine, Lahore Medical and Dental College, Lahore, PAK

**Keywords:** buprenorphine, methadone, oxycodone, fentanyl, chronic kidney disease (ckd), pain management in ckd patients, use of opioid in ckd patients, efficacy of opioids in chronic kidney disease, safety of opioids in chronic kidney disease, opioid use in chronic kidney disease

## Abstract

Patients with diminished renal function necessitate special care. In patients with chronic kidney disease (CKD), opioid analgesics should be prescribed based on the severity of renal insufficiency; this will determine treatment options at the beginning and throughout the management of pain in CKD patients. The dosage of hydrophilic drugs and drugs with active metabolites should be adjusted according to the severity of CKD, and the process of treatment should be monitored by modifying drug dosages as necessary for background and breakthrough pain. Patients with CKD may benefit from opioid analgesics that are lipophilic, such as methadone, fentanyl, and buprenorphine, as the first line; however, fentanyl is inappropriate for patients undergoing hemodialysis.

Opioid prescription in CKD patients is the subject of this systematic review, which aims to compare their safety and efficacy. This systematic review followed the Preferred Reporting Items for Systematic Reviews and Meta-Analyses (PRISMA) 2020 recommendations. Using three databases (PubMed, ScienceDirect, and Google Scholar), we collected and reviewed articles, including literature reviews, randomized control trials (RCTs), and systematic reviews published between 1980 and 2022, to enable us to gather enough valuable data on this rare topic. After applying appropriate filters, a total of 109 results were obtained. They were further screened and subjected to quality assessment tools, which finally yielded 11 studies included in this systematic review. This consisted of two RCTs, two systematic reviews, and seven narrative reviews.

This review focused on the safety and appropriate use of opioids in patients with CKD. The accumulation of morphine and codeine metabolites may result in neurotoxic side effects. Hydromorphone and oxycodone are considered safe to administer but require careful adjustments in dosage. Common comorbidities among patients with CKD may amplify opioid-related adverse effects.

## Introduction and background

Compounds classified as analgesics have various structural characteristics, modes of action, pharmacokinetic profiles, and analgesic potencies. They are separated into opioids and nonopioid medicines. Opioid analgesics are among the substances most frequently used during anesthesia and in the postoperative period to manage chronic pain in patients with and without cancer [1]. The choice of medication is made based on the mechanism causing the pain, but the patient's comorbid conditions must also be considered. Chronic kidney disease (CKD) is a comorbidity among them. Although opioids do not directly harm the kidneys, they can build up and cause renal failure. This is why it is crucial to routinely check on renal function in people who have used analgesics for a long time and examine any symptoms that patients describe [2].

Approximately 30 million people in the US currently suffer from chronic renal disease. Women are more likely than men to have CKD, which is indicated by an estimated glomerular filtration rate (eGFR) of less than 60 ml/min/1.73 m2 for more than three months [2,3].

The type and length of opioid prescriptions for patients with CKD and end-stage renal disease have been prescribed according to national prescribing patterns for the general population [4]. However, due to changes in pharmacokinetics and pharmacodynamics brought on by CKD, these prescriptions may be inappropriate for patients with CKD. A safe opioid prescription is essential because of the increased risk of side effects such as nausea, disorientation, constipation, drowsiness, respiratory depression, and myoclonus [5].

In affluent nations, the number of patients passing away from chronic renal illnesses is rising quickly. The significant symptom control requirements of these patients are becoming more widely recognized [6]. Pain is a widespread issue that has received inadequate attention and care. It is challenging to manage, mainly because impaired renal function restricts the use of drugs. Clinical suggestions are required to assist renal and palliative care experts in offering efficient pain management [7].

Patients with CKD have a heavy load of pain and symptoms. Concern over the hazards associated with opioid use and prescription is growing concurrently in patients with CKD. Nephrologists encounter challenging clinical scenarios more frequently that call for additional pain assessment and management, which may involve the use of opioids [8]. We discuss current strategies for managing pain with opioids in CKD, emphasizing kidney-specific issues. This entails a detailed assessment of the patient's pain level and treatment objectives. Following that, we review factors to consider before starting an opioid medication, ways to lower the associated risks, and suggest best practices for managing opioid use in CKD. Nephrologists can gain a comprehensive awareness of important patient issues, laying the groundwork for comprehending the effects of opioid usage with safety [9].

When opioid medications are required for pain management in patients with poor renal function, complications may arise. When a person has renal failure, their metabolism and pharmacokinetics are changed in a big way. This could lead to unpleasant side effects from the buildup of the original drug and its active metabolites [10]. The best way to use the most commonly used opioid analgesics to manage pain in individuals with renal impairment is presented and discussed in this study.

Numerous kidney lesions that have been well documented have been linked to intravenous drug usage. Recently, researchers discovered cases of opioid intravenous injection-associated granulomatous glomerulonephritis. However, this renal lesion also featured extensive tubulointerstitial involvement. In addition to being seen in the tubular basement membranes and glomeruli under electron microscopy, fibrillar deposits are not connected to immunoglobulin (Ig) deposition and lack the staining features of amyloid. This is a brand-new type of non-Ig-associated fibrillary glomerulopathy with opioid-related pathogenesis [11-14]. Finally, renal failure is a frequent finding in advanced cancer; as a result, oncologists frequently treat CKD patients. Opioids remain the mainstay of managing severe chronic pain in cancer patients; therefore, careful molecular selection is essential to prevent negative side effects. [15]

This systematic review aims to summarize the safety and efficacy of opioid prescriptions for patients with CKD.

## Review

Methods

Opioid prescription in CKD patients is the subject of this systematic review, which aims to compare their safety and efficacy. This systematic review followed the Preferred Reporting Items for Systematic Reviews and Meta-Analyses (PRISMA) 2020 recommendations [16].

Eligibility criteria

Studies in the English language, human studies, and free full-text articles published in the last 42 years (1980-2022) were selected as eligible for the review in order to collect sufficient information on this uncommon topic. Additional inclusion criteria include articles focusing on the prescription of opioids for CKD in patients of all ages. The criteria for exclusion included animal studies, gray literature, and paid articles.

Databases and search strategy

Through PubMed Central (MEDLINE), Google Scholar, and ScienceDirect, a search was conducted. Keywords and Medical Subject Heading (MeSH) terms were utilized to identify potentially relevant articles discussing the prescription of opioids to patients with chronic kidney disease. Terms like 'analgesics", "opioids", "administration and dosage", "opioid adverse effects", "opioid metabolism", "analgesic poisoning", "analgesics or opioids therapeutic use", "opioid toxicity and urine", "chronic renal insufficiency", "chronic renal disease", "chronic renal failure", "renal pathology", and "renal physiology" were searched for. The Boolean method was used to search through the various databases by combining keywords and MeSH terms. This systematic review's search strategy is detailed in Table 1.

**Table 1:** Details of the search strategy used in this systematic review

DATABASE	SEARCH STRATEGY	FILTERS APPLIED	RESULTS
PubMed	("analgesics opioid"[Pharmacological Action] OR "analgesics, opioid"[MeSH Terms] OR ("analgesics"[All Fields] AND "opioid"[All Fields]) OR "opioid analgesics"[All Fields] OR "opioid"[All Fields] OR "opioids"[All Fields] OR "opioid s"[All Fields]) AND ("renal insufficiency, chronic"[MeSH Terms] OR ("renal"[All Fields] AND "insufficiency"[All Fields] AND "chronic"[All Fields]) OR "chronic renal insufficiency"[All Fields] OR ("chronic"[All Fields] AND "kidney"[All Fields] AND "disease"[All Fields]) OR "chronic kidney disease"[All Fields]).	Free full-text, human, English	72
ScienceDirect	Opioid use in chronic kidney disease	Open access and open archive	10
Google Scholar	Opioids and chronic kidney disease		27

The EndNote reference manager (Clarivate, Philadelphia, PA, USA) was used to group and alphabetize all references for duplicate removal. The records were initially evaluated based on the titles and abstracts, with irrelevant studies eliminated. After this, full-text articles were reviewed for further exclusion.

Results

Study Selection and Quality Assessment

**Figure 1:**
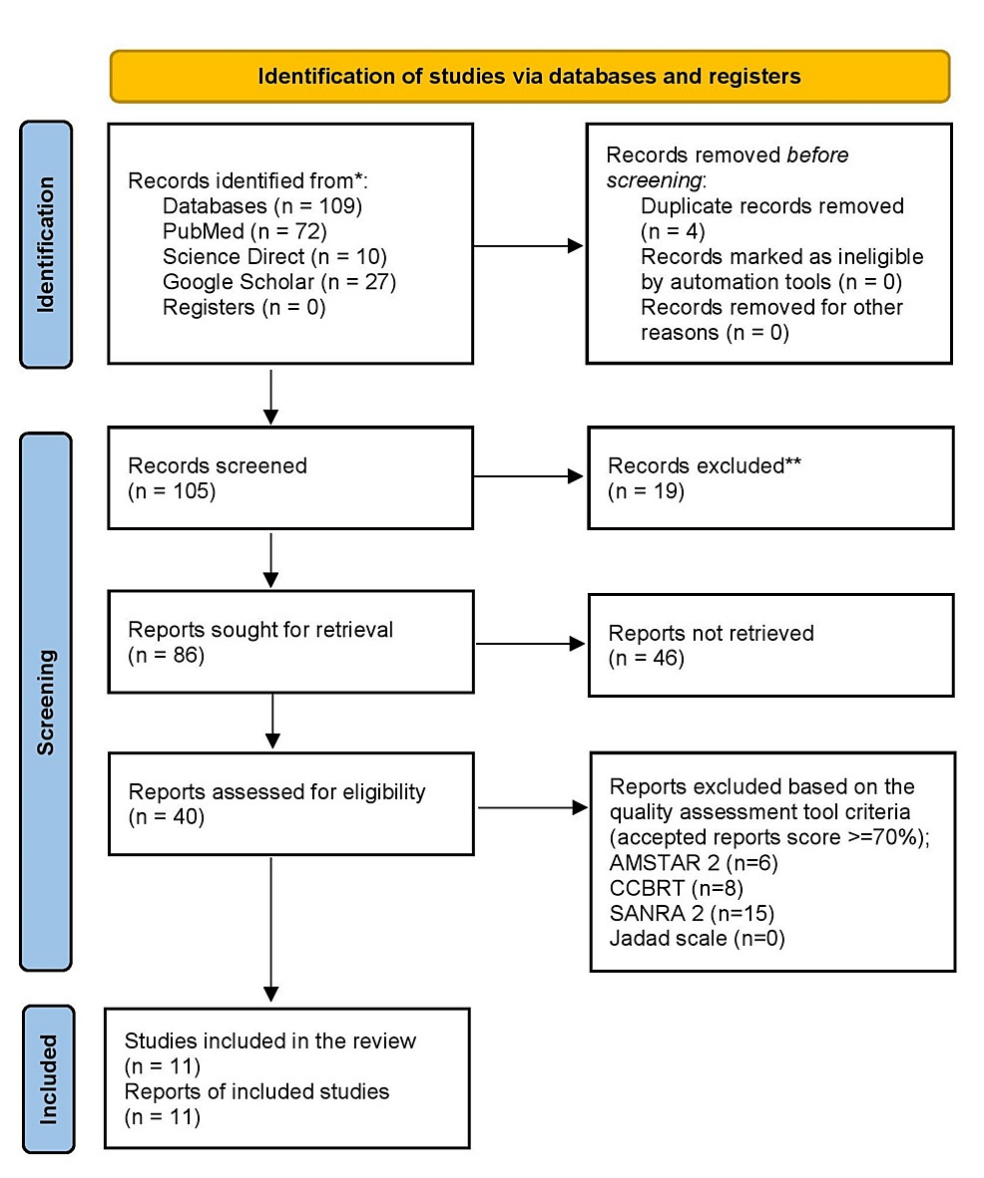
A flowchart demonstrating the selection process of the included articles as per the PRISMA guidelines PRISMA: Preferred Reporting Items for Systematic Reviews and Meta-Analysis; SANRA 2: Scale for the Assessment of Narrative Review Articles 2; AMSTAR 2: Assessment of Multiple Systematic Reviews 2; CCRBT: Cochrane Collaboration Risk of Bias Tool

One hundred and nine results were obtained using three databases (PubMed, ScienceDirect, and Google Scholar). These results were aggregated; four duplicates were eliminated, leaving 105. From the remaining 105 papers, 19 were eliminated due to extraneous titles and abstracts. The remaining 86 reports were exhaustively reviewed as full-text papers. Forty-six studies were excluded. The remaining 40 studies were then subjected to a quality assessment utilizing tools specific to each type of study. Eleven studies with quality assessment scores of greater than 70% were selected for inclusion in this review. There were two randomized controlled trials (RCTs), two systematic reviews, and eight narrative reviews. The final data collection date was May 31, 2023. A flowchart illustrating the identification and screening processes used to select the final articles for this review is depicted in Figure 1.

Table 2 below summarizes the quality assessment of the studies as well as the respective tools used.

**Table 2:** Details of the quality assessment tools used to assess the studies in this systematic review CCRBT: Cochrane Collaboration Risk of Bias Tool; RCT: randomized control trial; AMSTAR 2: Assessment of Multiple Systematic Reviews 2; PICO: population, intervention, comparison, and outcomes; RoB: risk of bias; SANRA 2: Scale for the Assessment of Narrative Review Articles.

Quality assessment tool	Type of study	Items and their characteristics	Total score	Accepted score (>70%)	Accepted studies
AMSTAR 2	Systematic reviews	Sixteen items: (1) Did the research questions and inclusion criteria for the review include the components of PICO? (2) Did the report of the review contain an explicit statement that the review methods were established prior to the conduct of the review, and did the report justify any significant deviations from the protocol? (3) Did the review authors explain their selection of the study designs for inclusion in the review? (4) Did the review authors use a comprehensive literature search strategy? (5) Did the review authors perform study selection in duplicate? (6) Did the review authors perform data extraction in duplicate? (7) Did the review authors provide a list of excluded studies and justify the exclusions? (8) Did the review authors describe the included studies in adequate detail? (9) Did the review authors use a satisfactory technique for assessing the risk of bias (RoB) in individual studies that were included in the review? (10) Did the review authors report on the sources of funding for the studies included in the review? (11) If meta-analysis was justified, did the review authors use appropriate methods for the statistical combination of results? (12) If a meta-analysis was performed, did the review authors assess the potential impact of RoB in individual studies on the results of the meta-analysis or other evidence synthesis? (13) Did the review authors account for RoB in individual studies when interpreting/discussing the results of the review? (14) Did the review authors provide a satisfactory explanation for and discussion of any heterogeneity observed in the results of the review? (15) If they performed quantitative synthesis, did the review authors carry out an adequate investigation of publication bias (small study bias) and discuss its likely impact on the results of the review? (16) Did the review authors report any potential sources of conflict of interest, including any funding they received for conducting the review? Scored as Yes or No. A partial Yes was considered a point.	16	12	King et al. (2011) [9], Sande et al. (2017) [10]
CCBRT	RCT	Seven items: random sequence generation and allocation concealment (selection bias), selective outcome reporting (reporting bias), other sources of bias, blinding of participants and personnel (performance bias), blinding of outcome assessment (detection bias), and incomplete outcome data (attrition bias). The bias was assessed as low-risk, high-risk, or unclear.	7	5	Benyamin et al. (2008) [2]. Wu et al. (2015) [12]
SANRA 2	Narrative review	Six items: justification of the article’s importance to the readership, statement of concrete aims or formulation of questions, description of the literature search, referencing, scientific reason, and appropriate presentation of data. Scored as 0, 1, or 2.	12	9	Soltysik et al. (2022) [1], Schieppati et al. (2005) [3], Murtagh et al. (2007) [4], Lu et al. (2021) [5], Coluzzi et al. (2020) [8], Niscola et al. (2010) [14], Dean M (2004) [15]

The studies included in this systematic review are summarized in Table 3.

**Table 3:** The studies included in this systematic review SANRA: Scale for the Quality Assessment of Narrative Review Articles; AMSTAR 2: Assessment of Multiple Systematic Reviews 2

First author, year	Report type	The quality assessment tool used	score
Soltysik et al. (2022) [1]	Review article	SANRA	11
Benyamin et al. (2008) [2]	Randomized control trial	Jadad scale	7
Schieppati et al. (2005) [3]	Review article	SANRA*	10
Murtagh et al. (2007) [4]	Review article	SANRA	10
Lu et al. (2021) [5]	Review article	SANRA	10
Coluzzi et al. (2020) [8]	Review article	SANRA	10
King et al. (2011) [9]	Systematic review	AMSTAR 2**	13
Sande et al. (2017) [10]	Systematic review	AMSTAR 2	13
Wu et al. (2015) [12]	Randomized control trial	Jadad scale	7
Dean M. (2004) [13]	Review article	SANRA	11
Niscola et al. (2010) [14]	Review article	SANRA	10
*SANRA checklist accepted score (>=70%): Minimum score 9 out of 12. ^Jadad scale accepted score (>=70%): Minimum score 6 out of 8; **AMSTAR 2 checklist accepted score (>=70%): Minimum score 12 out of 16			

Discussion

Chronic kidney disease is a collection of clinical signs and symptoms with abnormal laboratory test results caused by a chronic and progressive reduction in the number of nephrons that actively participate in renal activities. It also results in irreversible excretory and endocrine dysfunction of the kidneys [1].

Chronic pain affects CKD patients throughout their illness, not just those who receive dialysis in their later years. Significant impairment, a decline in health-related quality of life (HRQL), and a financial burden on the healthcare system are all highly correlated with chronic pain. Recognizing this, the nephrology community has encouraged routine screening and pain treatment to support patient-centered, outcome-oriented, high-quality healthcare. This includes a growing understanding that some CKD patients with severe discomfort can benefit from analgesics, including opioids. However, patients are more likely to experience drug-related issues because of the changed pharmacokinetics and pharmacodynamics of most analgesics in CKD, and there are still valid concerns about the safety of opioids and other painkillers for chronic pain in CKD [11].

There is more appropriate and improper analgesic use as pain frequency and intensity rise, which could put patients at risk for analgesic-related side effects. Several crucial considerations should be considered when dealing with CKD patients who experience chronic pain to reduce potential harm. An extensive treatment plan with physical therapy, and psychological and behavioral pain management strategies should be employed with analgesics. The best way to do this is through a multidisciplinary process and the evaluation of aggravating symptoms like sadness and anxiety. A complete approach to pain should also include negotiating reasonable pain treatment goals and patient education. The choice of analgesic when contemplating chronic analgesic therapy is crucial, considering the severity of the renal failure, interactions with other drugs being coadministered, and comorbidities. The dangers and advantages for specific patients must also be carefully considered. With careful titration, acetaminophen and/or low-dose adjuvant medicine, such as gabapentin, can effectively manage pain. Patients with CKD with significant pain are unresponsive to non-pharmacological or nonopioid therapy and impair physical function, and HRQL may be candidates for opioids. Clinical data indicates that most CKD patients with severe pain can usually benefit from low doses [12].

Opioid Pharmacokinetics

Opioid pharmacokinetics are significantly altered in patients with impaired renal function, depending on factors such as the volume of distribution, active metabolites, and the lipophilicity and hydrophilicity of opioids. Parent pharmaceuticals and their metabolites may be eliminated less efficiently (via glomerular filtration (GFR) or renal tubular excretion). Changes in gastric juice pH or gastrointestinal motility, which opioids and medications with spasmolytic effects may cause, can also affect the bioavailability of drugs. Changes in the distribution, metabolism, and protein binding of pharmaceuticals and their escape distribution may also occur in patients with CKD. They frequently have comorbid conditions, such as cachexia and hypoproteinemia, which alter the volume of drug distribution. Opioids vary in terms of efficacy and tolerance in individuals with renal failure. As a result of changes in drug pharmacokinetics in CKD patients, their use may increase the incidence of adverse effects, and this effect varies depending on the opioid used [9].

Opioid Metabolism

Due to decreased clearance and a higher buildup of the parent analgesic and active metabolites, patients with kidney failure are more likely to have adverse opioid side effects. Dialysis may also eliminate analgesics, impacting the treatment's analgesic outcomes.

The enzyme system(s) that the opioid is metabolized by, along with the patient's genetic makeup and underlying health issues (most notably, kidney or liver illness), are what essentially influence the risks of toxicity, inadequate opioid response, and drug interactions. Opioid metabolism occurs mainly in the liver, while the kidneys excrete the metabolites and various amounts of the parent substance. Both inert and some of the active metabolites, which may be more potent than the parent substance, are produced due to opioid metabolism. Patients with impaired renal function will accumulate these to varying degrees, and they often have a little therapeutic window between analgesia and toxicity. Opioid drugs must be chosen carefully, and knowledge of opioid metabolism is crucial in making this choice [17].

The Effects Of Dialysis

The stability of opioids during dialysis differs between opioids. Dialyzed opioids may require supplemental administration during hemodialysis (HD) or after HD if needed, and these patients may also be at an increased risk for withdrawal symptoms following dialysis. Poorly dialyzed opioids have a longer-lasting analgesic effect. However, the risk of toxicity increases with the accumulation of the parent substance and active metabolites. The ability of dialysis to eliminate any substance depends on several variables. Lower molecular weight, greater water solubility, lower protein binding, and a lower volume of distribution are factors that promote opioid dialyzability [4].

Side Effects of Opioids and Their Management in CKD

In managing chronic pain, the tolerability of opioids is frequently affected by side effects such as urinary retention, drowsiness, dizziness, vomiting, nausea, and constipation.

Respiratory depression is the most dreaded adverse outcome following opioid administration. It can present with respiratory depression, a life-threatening condition, but this is not common in patients who have been using opioids to manage their chronic pain, and it is primarily associated with preexisting conditions, which can increase the risk of inadequate accidental dosages. Naloxone is an opioid receptor antagonist that rapidly restores normal breathing following an opioid overdose. In emergencies, when a patient's breathing has slowed or ceased due to an opioid overdose, 0.4mg to 2mg of naloxone administered intravenously can be repeated every two to three minutes when the respiratory depression reversal is not obtained.

Some opioids have a longer half-life than naloxone; as a result, it would be necessary in such cases to administer multiple doses of naloxone while monitoring the vital signs until the goal of reversal is achieved [18].

The most enduring and prevalent adverse effect of opioids is opioid-induced constipation. This is due to gastrointestinal motility being controlled by the widely distributed opioid receptors. Opioids physiologically control water absorption. It limits water secretion by acting in the submucosal plexus of the mu-opioid receptor (MOR) in the enteric nervous system of the gastrointestinal lumen. Patients do not develop a tolerance to opioid-induced constipation (OIC), so long-term treatment is required. To manage this side effect, in the last decade, the introduction of peripherally acting mu-opioid receptor antagonists (PAMORA) in addition to conventional laxatives has been useful. These compounds act antagonistically exclusively in the intestinal nervous system because they cannot cross the blood-brain barrier. Few data exist on the use of PAMORA in CKD patients. A single 25mg dose of naloxegol, a pegylated derivative of naloxone, has shown great tolerance in patients with chronic kidney disease. Due to its modest molecular weight, HD efficiently clears naloxegol. However, the initial dose for patients whose renal impairment is moderate to severe should be reduced to 12.5mg. In contrast, naldemedine, a derivative of naltretion, is administered at the standard dose of 0.2mg every day in patients with CKD, comparable to patients with normal kidney function. No dose adjustments are necessary [8].

In a subgroup analysis of patients with renal impairment, a daily dose of 0.2mg was effective and safe, consistent with the overall population. Naloxegol and naldemedine administered orally are metabolized through cytochrome P450 system pathways (CYP450). Chronic kidney disease patients may downregulate the expression of the cytochrome P450 system (CYP3A4) due to the accumulated uremic toxins. The CYP3A4 is the primary enzyme responsible for the metabolism of PAMORA. Therefore, inhibitors or inducers of CYP3A4 may have a significant effect on naldemedine and naloxegol. Their concurrent use is contraindicated. On the other hand, methylnaltrexone is not metabolized by CYP450, but in patients with a creatinine clearance of 60 mL/min, a reduced dose of 12mg to 8mg should be considered. [19].

Pharmacological Mechanism

**Figure 2:**
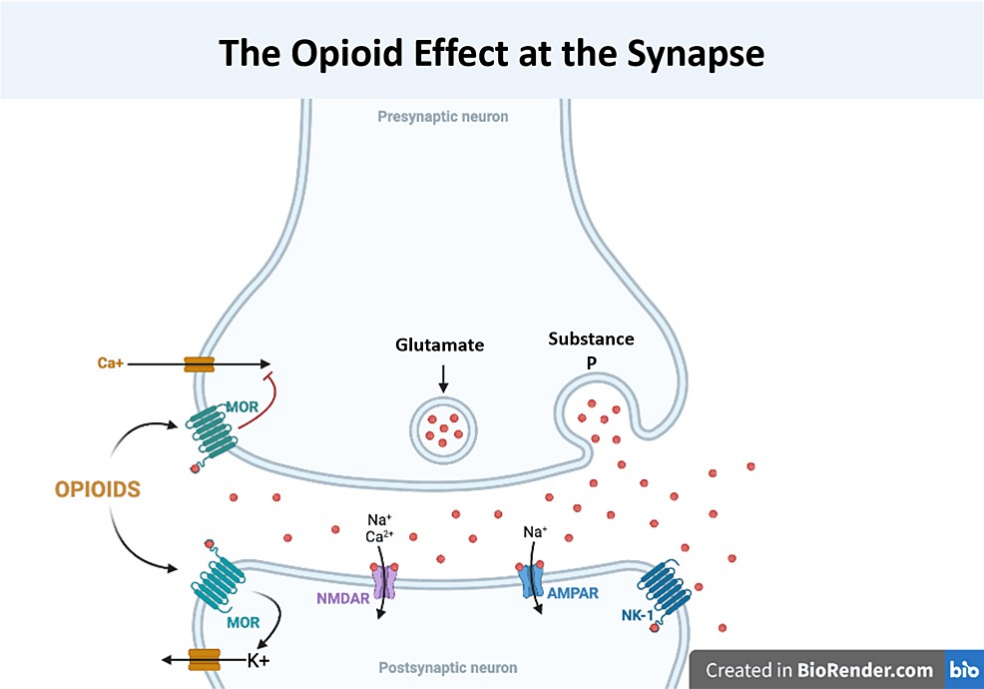
The opioid effect at the synapse This illustration is an original creation of the authors of this manuscript MOR: mu-opioid receptor; NMDAR: N-methyl-D-aspartate receptor; AMPAR: α-amino-3-hydroxy-5-methyl-4-isoxazolepropionic acid receptor; NK-1: neurokinin-1 receptor

The endogenous physiologic function of opioids acts as an analgesic. This is carried out by endorphins acting on opioid receptors. The opioid receptors are made up of four coupled G-protein receptors with seven transmembrane domains that have been identified. Those are as follows: mu-opioid receptor (MOR), delta-opioid receptor (DOR), nociceptive opioid receptor (NOR), and kappa-opioid receptor (KOR). There are four main endogenous ligand families, which include enkephalins, endorphins, nociceptin/orphaninFQ, and dynorphins. By interacting with potassium and calcium channels, opioids inhibit neurotransmission. Calcium influx is inhibited on primary afferent fibers (PAF) in the first-order neuron, leading to the subsequent release of excitatory neurotransmitters, including substance P and glutamate. Opioid receptors are also expressed by second-order neurons in the dorsal horn; they activate the rectifying potassium channel. As a result, it stimulates postsynaptic hyperpolarization. These mechanisms result in an efficient modulation of pain transmission and clinically appropriate analgesia [20]. Figure 2 illustrates a representation of the effect of the opioid on the synapse.

Buprenorphine

Buprenorphine appears especially beneficial for treating severe chronic pain in CKD patients. First, it does not produce active metabolites that can accumulate and promote toxicity. The liver metabolizes buprenorphine to form two metabolites that are excreted by the bile: norbuprenorphine-3-glucuronide (N3G) and buprenorphine-3-glucuronide (B3G) [21].

Several studies examined whether renal dysfunction could influence buprenorphine clearance and found that this parameter was comparable in patients with impaired renal function and normal renal function [22].

Furthermore, the efficacy and safety of buprenorphine in patients with chronic kidney disease without dose adjustment make it well-suited for managing pain in CKD patients [23].

The cytochrome CYP3A4 converts buprenorphine to norbuprenorphine, which is weaker than buprenorphine but has analgesic properties. These compounds undergo glucuronidation and are excreted. The gastrointestinal tract changes by about 70%-80%. About 10%-30% is changed to glucuronide and norbuprenorphine in the urine. To maintain the therapeutic concentration of the drug in the blood, the transdermal form of this drug is preferred to avoid being eliminated by the first-pass effect. Buprenorphine can be administered safely to patients with impaired renal function and hemodialysis patients in the dose range of up to 70 g/h because its pharmacokinetic properties are unaffected [15].

Fentanyl

Fentanyl is a complete opioid receptor agonist. It swiftly crosses the blood-brain barrier due to its high lipophilicity and low molecular weight and binds to the receptor in the central nervous system. It is metabolized via cytochrome CYP3A4 to norfentanyl. Seventy-five percent is excreted as an inactive metabolite in the urine and 9% in the feces. About 10% of the drug is excreted in urine unchanged [1].

In patients with CKD and compensated hepatic disease, the half-life of fentanyl is not significantly prolonged, whereas in patients with CKD, this parameter doubles after the first pass through the liver. About 90% of fentanyl is converted to an inactive metabolite by the liver. The high hepatic clearance is dependent on the liver's blood supply and degree of extraction as it travels through the liver [24].

Fentanyl administered orally is absorbed through the portal system into the bloodstream. As a result, the entire dose first goes through the liver. During the first pass, 60% is inactivated, which makes maladministration ineffective. In the case of transmucosal administration, the bioavailability of the drug fluctuates according to its form (intranasal, sublingual, or buccal) [9].

The clearance of biotransformed fentanyl into inactive metabolites in patients with CKD varies. Such patients frequently experience excessive drowsiness and are at a higher risk for respiratory depression. Fentanyl is a relatively safe opioid for CKD patients [24-26].

Methadone

Methadone is a synthetic, receptor-agonist opioid medication. It is also an antagonist of N-methyl-D-aspartate (NMDA) receptors and an inhibitor of serotonin and norepinephrine reuptake (SNRI). Only 1% of methadone is found in the blood, the rest forming a reservoir in the tissues, resulting in a very long elimination time (approximately 15-60 hours) [24].

Methadone is converted to inactive metabolites in the intestinal wall and liver. Around 10%-45% is excreted in the gastrointestinal tract, and 25%-50% is excreted via the kidneys.

The elimination time of methadone from the body is unaffected by impaired hepatic or renal function. In cases of anuria, the medicine is excreted almost exclusively as pyrolidine via the gastrointestinal tract. Methadone can be administered safely to CKD patients. A half-dose should be considered in patients with reduced GFR of 10-15 ml/min and a serum creatinine level above 8 mg/dL (700 mmol/L) [1].

Methadone appears safe in CKD patients since it is eliminated in feces. Plasma concentrations should be compared in these patients to those observed in healthy controls [11, 13, 27].

Hydromorphone

Hydromorphone is five times more potent than morphine. It does not cause central nervous system (CNS) depressant activities because it lacks the metabolite 6-glucuronide. A 2008 prospective observational study revealed that hydromorphone does not accumulate, likely due to its rapid conversion to the hydromorphone-3-glucuronide (H3G) metabolite. Indeed, H3G can accumulate in patients with low GFR (10 mL/min/1.73 m2), resulting in myoclonus and delirium [28, 29].

Furthermore, H3G can accumulate between dialysis sessions, likely resulting in increased sensory-type pain and decreased analgesic duration. Based on experimental evidence, hydromorphone is free of adverse effects when administered at standard doses to patients with CKD and is relatively safe for dialyzed patients. However, patients should be monitored closely [30].

Morphine

Morphine is a mild and pure opioid receptor agonist. It is metabolized in the liver, intestinal wall, central nervous system, and kidneys via conjugation with glucuronide acid. About 60% to 80% is metabolized to morphine-3-glucuronide (M3G), 10% is glucuronidated to morphine-6-glucuronide (M6G), and 5% is metabolized to normophine. Ten percent is excreted in the urine unchanged. A small amount of morphine is metabolized to codeine [1].

Patients receiving morphine intravenously have higher concentrations of morphine than patients receiving oral morphine administration. This is due to the ratio of M3G to M6G after the first-pass effect. The analgesic effects of M6G are 10-60 times more potent than those of morphine, while M3G's neurotoxic effects cause opioid hyperalgesia.

Up to 30% of morphine is metabolized outside the liver under normal conditions. Creatinine clearance directly influences the excretion of morphine glucuronides. About 90% of conjugated morphine is excreted in urine, with the remainder in sweat, bile, and milk [11].

The kidneys eliminate morphine and its active metabolite glucuronides, which may result in accumulation, adverse effects, and unstable analgesia. Therefore, it is not advised for patients with renal failure. The morphine glucuronide half-life is extended from four to 14 to 119 hours in patients with CKD. Furthermore, the accumulation of metabolites occurs [13].

In addition to the accumulation of M6G in renal failure, glucuronides can be hydrolyzed to their parent compound. Uremia can make CNS symptoms like decreased level of consciousness, apathy, drowsiness, impaired ability to think in a complex way, and impaired concentration worse. Patients with CKD can experience more adverse effects due to the increased permeability of morphine through the blood-brain barrier, leading to sedation, respiratory depression, nausea, and vomiting. The drug dosage should be increased from four to eight hours to reduce the risk of their occurrence. [31].

Oxycodone

Oxycodone is a more potent analgesic and has a quicker onset of action. It is commonly used in the management of chronic pain. It has minor side effects like mental disarray and hallucinations. [32].

Patients with CKD must carefully monitor their oxycodone prescriptions. Ten percent of oxycodone is excreted in urine unchanged. Multiple metabolites are generated via hepatic metabolism, which includes nor-oxycodone and oxymorphone (further metabolized to noroxycodone). These metabolites may accumulate in CKD patients due to renal impairment in eliminating oxycodone. Patients with CKD have peak plasma concentrations that are 50% higher than those of healthy individuals [33].

A minor amount of oxycodone is eliminated via feces. [34].

The oxycodone half-life is prolonged in CKD patients. Therefore, a lower dose should be used in CKD patients. However, the effect of oxycodone is mainly dependent on the parent drug [1].

Tramadol

Concerning the use of the "weak opioid" tramadol, it is pertinent to note that the kidneys excrete 90% of the drug. In CKD patients, the half-life of tramadol increases by up to twofold [35]. Thirty to 100mg of tramadol, with a maximum daily dose of 200mg, should be considered in treating patients with a creatinine clearance of 30 ml/min [36].

Two enantiomers make up the racemic composition, which is tramadol. Both enantiomers influence the monoamine system, but only O-desmethyltramadol, the (+) enantiomer, affects opioid receptors. O-desmethyltramadol is the main tramadol metabolite produced by cytochrome P4502D6 enzyme (CYP2D6). The CYP3A4 produces inactive N-desmethyltramadol as the other metabolite [11].

However, there is a higher risk of convulsions in patients with renal failure. The kidneys and a minor amount of the digestive tract eliminate up to 90% of tramadol and its metabolites. Therefore, it seems reasonable to increase the time between drug administrations in patients with CKD to eight or 12 hours [37].

In the case of CKD patients, the administration of tramadol is contraindicated when the GFR falls below 10 ml/min. Patients with CKD should not be administered the controlled-release formulation of tramadol [1].

Codeine

It is a 10-fold weaker opioid receptor agonist than morphine. Glucuronidation is the main metabolic pathway 80% of morphine undergoes to form codeine-6-glucuronides, while 10% of codeine is metabolized to norcodeine by CTP3A4 via N-demethylation. Less than 10% of codeine undergoes O-demethylation by the CYP2D6 to become morphine. The kidney then almost exclusively eradicates codeine and its metabolites from the body, primarily conjugated with glucuronic acid. About 17% of metabolites are excreted in urine. The primary analgesic mechanism is the biotransformation of codein to morphine.

Wide variations exist in the CYP2D6 isoenzyme's activity. Up to 5%-10% of Caucasians lack this isoenzyme, rendering them codeine-insensitive (weak metabolizers). However, in a small percentage of the population (ultrarapid metabolizers), this gene is overexpressed, leading to a rapid conversion of codeine to morphine [11].

The kidneys excrete codeine and its metabolites, which accumulate in CKD patients and may result in toxic symptoms. The dosage of codeine should be reduced by 50% and carefully titrated. Due to the conversion of codeine to morphine, which is contraindicated in this patient population, codeine should not be administered to those with CKD [13].

Clinical outcomes and dosage considerations in CKD are shown in Table 4 below.

**Table 4:** Clinical outcomes and dosage considerations in CKD Vd: volume of distribution; WS: water solubility; PPB: plasma protein binding; MW: molecular weight; N3G: norbuprenorphine-3-glucuronide; B3G: buprenorphine-3-glucuronide; H3G: hydromorphone-3-glucuronide; H6G: hydromorphone-6-glucuronide; C6G: codeine-6-glucuronide; AUC: area under the curve; CYP2D6: cytochrome P450 2D6 enzyme; M3G: morphine-3-glucuronide; M6G: morphine-6-glucuronide; CYP3A5: cytochrome P450 3A5 enzyme; CYP2C8: cytochrome P450 2C8 enzyme; CYP2C19: cytochrome P450 2C19 enzyme; CYP2B6: cytochrome P450 2B6 enzyme; CYP2C18: cytochrome P450 C18 enzyme; CYP2C9: cytochrome P450 2C9 enzyme; UGT2B7: UDP glucuronosyltransferase 2B7 enzyme; UGT2B4: glucuronosyltransferase 2B4 enzyme

Opiod	Metabolism	Elimination	Clinical use	Dosage consideration
Buprenorphine (Davis M, 2012) [38]	Extensive hepatic first-pass metabolism. Norbuprenorphine is produced via CYP3A4 and CYP3A5 in phase 1 metabolism. Phase 2 metabolism via glucuronidation to inactive substances B3G and N3G.	Metabolites are eliminated predominantly through feces. 10%–30% of the dose is eliminated in the urine.	Safer profile. No dosage modification is necessary at any stage of CKD.	The patch is considered safe (Niscola et al., 2010) [14].
Fentanyl citrate (Joh et al., 1998) [39]	Significant hepatic metabolism to inactive metabolites. 99% of norfentanyl undergoes phase 1 metabolism via CYP3A4 to become norfentanyl.	7% unchanged in the urine and 1% unchanged in the excrement.	Safer profile. No accumulation is clinically significant in CKD.	Appears safe, but contemplate a dose reduction with long-term use (Niscola et al., 2010) [40].
Methadone (Furlan et al., 1999) [40]	Extensive hepatic metabolism in the first pass into inactive metabolites. N-demethylation CYP2C19, CYP3A7, and CYP2C8 preferentially metabolize (R)-methadone; CYP2B6, CYP2D6, and CYP2C18 preferentially metabolize (S)-methadone; CYP3A4 does not have an enantiomer preference.	After extensive biotransformation, excreted through the feces and urine; 20% of urine excreted unchanged.	Due to its long half-life, it is contraindicated to be used with other analgesics. the toxicity may be delayed in CKD patients requiring a reduction in dosage.	Dosage, despite the fact that it appears relatively safe, requires a specialized setting with a competent and experienced team (Pergolizzi et al., 2008) [41].
Hydromorphone (Dean M., 2004) [13]	Extensive hepatic first-pass metabolism (62%). Glucuronidation via UGT2B7 to H3G without analgesic activity (possibly causes neuroexcitation, agitation, and confusion). Minor phase 1 metabolism to norhydromorphone via CYP3A4 and CYP2C9.	Mostly eliminated as H3G in the urine. 7% of the urinalysis is unchanged. 1% is excreted unchanged in the feces.	H3G builds up between dialysis sessions. Easy to dialyze: HD removes 60%. Subsequent to sudden decreases in opioid concentrations, withdrawal symptoms may develop. There may be a need for supplemental dosing after dialysis.	Decrease Dosage and use with caution by monitoring symptoms closely (Razaq et al., 2007) [42].
Morphine (Pergolizzi et al., 2008) [41]	90% of phase 2 metabolism occurs via glucuronidation by UGT2B7 to: -M3G without analgesic activity, but possibly neurotoxic -M6G that has analgesic properties. Minor conversion of morphine to normorphine.	70%–80% are eliminated in the urine. 10% excreted unchanged in the feces and 10% unchanged in the urine.	Not recommended due to the accumulation of metabolites. To avoid in CKD patients.	Do not use (Mercadante et al., 2002) [43].
Oxycodone (Pergolizzi et al., 2008) [41]	Phase 1 metabolism: -through CYP3A4 and CYP3A5 (N-demethylation) to noroxycodone, followed by CYP2D6 to noroxymorphone -through CYP2D6 (O-demethylation) to oxymorphone, followed by CYP3A4 to noroxymorphone.	Primarily eliminated via urine: 23 % free noroxycodone 10% conjugated oxymorphone 9% free and conjugated oxycodone and less than 1% oxymorphone.	HD eliminates oxycodone and its active metabolite, noroxycodone. Oxycodone has been administered to patients who are dependent on dialysis, and it has been well tolerated without the need for opioid compensation.	Reduce dosage, adjust administration interval, or discontinue use (Foral et al., 2007) [44].
Tramadol (Gardner et al., 2000) [45]	Extensive hepatic first-pass metabolism phase 1 of metabolism: -N-demethylation by CYP3A4 and CYP2B6 to Ndesmethyl-tramadol (M2) -conversion via CYP2D6 (O-demethylation) to odesmethyltramadol (M1).	90% is eliminated in the urine (30% unchanged); 10% is eliminated in the feces.	Hemodialysis results in a significant removal. After an HD session, it may be necessary to reduce.	Decrease dosage, increase dose interval, and cautious patient monitoring (Niscola et al., 2010) [14].
Codeine (Dean M., 2004) [13]	80% of phase 2 metabolism is glucuronidation by UGT2B7 and UGT2B4 to C6G. Phase 1 metabolism: -through CYP3A4 (N-demethylation) to norcodeine (10%) devoid of analgesic properties -via CYP2D6 (O-demethylation) to morphine (5%-10%).	90% excreted by the kidneys and 10% excreted unchanged in the urine.	Hemodialysis results in a significant removal. Re-dosing may be required following an HD session.	In some patients with uremia receiving multiple doses, the Dosage may need to be adjusted, or the medication should be avoided. (Guay et al., 1988) [46].

The safest opioids for patients with advanced CKD are described, along with their pharmacokinetic properties. Fentanyl, methadone, hydromorphone, and buprenorphine could be helpful in managing pain in CKD patients due to their minimal alteration in kinetics. They may have a long-lasting analgesic effect during hemodialysis. Hydromorphone is advantageous because it does not undergo phase 1 metabolism, thereby averting the complications of drug-drug interactions and toxicity during metabolism with CYP2D6 and CYP34A. Both methadone and fentanyl lack active metabolites. In addition to CYP3A4, several CYP enzymes are required to metabolize methadone, making the potential for drug-drug interactions complex. [10].

Limitations

The studies included in this systematic review had some limitations. This study was limited to free full-text articles published from 1980 to 2022; due to this, we may have overlooked articles that were published before 1980. Additionally, publications in languages other than English were rejected during the screening process; studies in which animal models have been used have been excluded from the review. Despite these limitations, the results of this systematic review provide valuable insights into the safety and efficacy of opioid use in CKD patients.

## Conclusions

Pain is common and difficult to treat among patients with kidney disease. The risks and benefits of various pharmacological and non-pharmacological therapeutic options must be carefully evaluated, continuously monitored, and shared with patients to manage pain effectively. Long-term treatment with opioids that are full agonists is associated with significantly more significant adverse effects in this population. The reduction of the dosage of opioids and prolonging the intervals between dosing should be considered in each case at various disease stages based on GFR. If extended-release medication does not cause adverse effects in CKD patients, they should continue to be on the same regimen. Analgesic opioids that are lipophilic are regarded as secure. The treatment of preference appears to be transdermal administration of fentanyl or buprenorphine. Half the dose should be considered when initiating therapy, particularly in patients with advanced renal failure. In cases of inadequate analgesia, the optimal amount is determined using the same titration principles as in patients with normal renal function. Using opioid analgesics, including opioid receptor antagonists, in end-stage CKD requires renal replacement therapy.

Further research is needed to confirm the safety and efficacy of more opioids for managing pain in CKD patients. Future studies should be designed with larger sample sizes, more extended follow-up periods, and appropriate control groups to provide more robust evidence of the effectiveness and safety of opioid use in CKD patients. Additionally, studies evaluating the comparative efficacy of other opioids to the existing treatments mentioned in this systematic review would be valuable to help inform more treatment options decisions.
